# Vitamin C: A Comprehensive Review of Its Role in Health, Disease Prevention, and Therapeutic Potential

**DOI:** 10.3390/molecules30030748

**Published:** 2025-02-06

**Authors:** Adina Alberts, Elena-Theodora Moldoveanu, Adelina-Gabriela Niculescu, Alexandru Mihai Grumezescu

**Affiliations:** 1 Carol Davila University of Medicine and Pharmacy, 050474 Bucharest, Romania; adina-magdalena.alberts@rez.umfcd.ro; 2 National University of Science and Technology Politehnica Bucharest, 011061 Bucharest, Romania; moldoveanu.theodora99@gmail.com (E.-T.M.); adelina.niculescu@upb.ro (A.-G.N.); 3 Research Institute of the University of Bucharest—ICUB, University of Bucharest, 050657 Bucharest, Romania

**Keywords:** vitamin C, ascorbic acid, enzymatic cofactor, antioxidant, pro-oxidant effect health, scurvy, cardiovascular diseases, cancer, infections, chronic diseases

## Abstract

Since Albert Szent-Györgyi discovered it and it became used in treating scurvy, vitamin C has attracted interest in many studies due to its unique properties. It is an important cofactor in the synthesis of collagen and hormones, and it is involved in immunity, iron absorption, and processes requiring antioxidants. Thus, this review aims to emphasize the importance and usefulness of vitamin C in improving quality of life and preventing various diseases (e.g., chronic diseases, cardiovascular diseases, cancer) but also for its use in treatments against infections, neurodegenerative diseases, and cancer. Although the studies presented provide essential information about the properties of VIC and its beneficial effect on health, some studies contradict these theories. In this respect, further studies on larger samples and over a longer period are needed to demonstrate the therapeutic potential of this nutrient. However, VIC remains a necessary vitamin that should be consumed daily to maintain optimal health and prevent deficiencies that can lead to scurvy and its associated complications.

## 1. Introduction

Vitamin C (VIC), also known as L-ascorbic acid, is a well-known micronutrient necessary to maintain the human body’s overall health. Found in two main forms, namely, ascorbic acid (AA) and dehydroascorbic acid (DHA), VIC is a strong antioxidant and a cofactor for many enzymes, being involved in many biological functions, such as normal immune system functioning, carnitine and catecholamine metabolism, dietary iron absorption, and collagen biosynthesis [1,2,3,4]. Still, the body cannot synthesize it due to mutations in the L-gulonolactone oxidase (GLO) gene, which is responsible for its synthesis from L-gulono-1,4-lactones. Therefore, VIC intake is attributed to the high consumption of foods rich in ascorbic acid (e.g., tomatoes, potatoes, green leafy vegetables, berries, citrus fruits). VIC can be accumulated in different tissues (e.g., brain, liver, skeletal muscles, corneal epithelium, neutrophile, stem cells) via specific transporters, and it can enhance cellular functions [5,6,7,8].

VIC is important for realizing numerous biological functions as it is a potent antioxidant and a cofactor for enzymes present in the human body. VIC can neutralize reactive oxygen species (ROS) produced during normal metabolism, thereby preventing oxidative stress and cellular damage [2,6]. Additionally, in high doses, vitamin C can act as a pro-oxidant, a property that is being studied for its potential in cancer treatment [5,9].

VIC is also involved in the hydroxylation of collagen, the synthesis of carnitine and catecholamines, and the metabolism of tyrosine, folic acid, and tryptophan. VIC is also involved in the hydroxylation of proline, lysine, and glycine, which stabilizes collagen’s triple-helix structure. Furthermore, VIC promotes the conversion of cholesterol into bile acids, contributing to cholesterol homeostasis and reducing the risk of hypercholesterolemia and gallstone formation [2,8,10].

VIC also stimulates iron absorption at the intestinal level by reducing Fe^3+^ to Fe^2+^, making it more stable and contributing to anemia management [2,8,10]. In energy metabolism, VIC supports the synthesis of carnitine, which is responsible for transporting fatty acids into mitochondria [7,10]. Another essential role for VIC may be its ability to act as a cofactor for the enzyme dopamine-beta-hydroxylase, contributing to converting dopamine to norepinephrine, an essential neurotransmitter for the nervous system [2,10].

Thus, VIC can contribute significantly to maintaining long-term health. Although VIC deficiency is becoming increasingly common worldwide, even in developed countries, it is important to maintain an adequate intake to contribute to improved health and optimal immune, cardiovascular, and neurocognitive functions [11]. Since humans cannot synthesize VIC on their own, its dietary intake is important to prevent deficiency, being efficiently absorbed in small amounts. The main source of VIC is fresh fruits (e.g., kiwi fruit, citrus fruits) and vegetables (e.g., peppers, green leafy vegetables), while meat, cereals, and tubers are low in VIC. How these foods are eaten is also important, as high temperatures can destroy VIC during cooking [12].

Deficiency of VIC may also be associated with different factors, including environmental, socioeconomic, and physiological aspects. Populations in regions with limited access to fresh produce, such as Finland and Russia, show a higher deficiency prevalence. In contrast, areas like England and China, where fresh, VIC-rich foods are consumed regularly, exhibit lower prevalence, even during winter months. Socioeconomic status profoundly influences VIC intake, as individuals in disadvantaged circumstances often lack access to nutrient-rich diets. Vulnerable groups such as the elderly, children, and individuals with chronic conditions—including obesity, cardiovascular disease, diabetes, cancer, and HIV—are particularly at risk due to altered metabolic demands and suboptimal nutrient absorption. Lifestyle factors, including smoking and alcohol consumption, further deplete VIC levels due to increased oxidative stress [12,13].

Severe and prolonged VIC deficiency leads to scurvy, a condition that has affected people since ancient times. The disease has been historically linked with limited dietary access among sailors and soldiers in the 15th and 16th centuries. At present, scurvy is considered a rare disease in developed countries, but it can occur in vulnerable social environments, in alcohol consumers, and in people with severe psychiatric problems [13,14,15]. A 2023 study by Miraj et al. [16] demonstrated scurvy’s contemporary relevance in pediatric populations subsisting on processed diets devoid of fresh produce. Among 18 cases analyzed, lower limb pain, subperiosteal hemorrhages, and radiographic findings such as Frankel’s white line were predominant features, alongside gingival pathology and anemia. Symptomatic resolution occurred within 4 to 16 weeks following administration of 100–250 mg/day.

Based on these findings, efforts to combat VIC deficiency should prioritize increasing access to fresh produce and promoting public health initiatives targeting high-risk populations. While supplementation alleviates deficiency, its bioavailability remains lower compared to natural dietary sources of VIC. Consistent intake of approximately 200 mg/day through diet and supplements is crucial for sustaining adequate VIC levels, ensuring overall health improvement, and reducing the risk of deficiency-related complications [12,17,18].

The pathogenesis of scurvy is linked to deficient collagen synthesis due to the absence of VIC, impairing connective tissue integrity and capillary stability. Initial symptoms include fatigue, irritability, and appetite loss caused by impaired carnitine biosynthesis and fatty acid transport. Progression results in perifollicular hemorrhages, gingival hypertrophy, delayed wound healing, myalgia, muscle weakness, hyperkeratosis, weight loss, and hematological abnormalities. Advanced cases can culminate in sepsis-like syndromes, hypotension, and death [14,15,19,20,21,22,23,24].

To treat scurvy, researchers such as William Bally, Axel Holst, Theodor Frolich, and Sir Frederick Gowland Hopkins contributed to understanding how VIC works in the human body. This research led to the chemical formula for VIC, which was later recognized as ascorbic acid, and its importance in curing scurvy [25,26,27]. Albert Szent-Györgyi’s isolation of “hexuronic acid”, later termed ascorbic acid, illuminated its anti-scorbutic properties and earned him the 1937 Nobel Prize in Medicine [28,29].

Nowadays, VIC is no longer only used as a treatment for scurvy, but its benefits in various biological functions have also been recognized. Thus, VIC has been shown to have an extremely good antioxidant effect, protecting against oxidative stress, facilitating collagen synthesis, supporting skin health, enhancing immune function, improving wound-healing, and promoting iron absorption in the intestine. However, studies are continuing to explore its potential in dealing with some major diseases, such as cardiovascular disease, cancer, and age-related diseases [4,19,30,31,32]. Emerging research also highlights the role of VIC in maintaining oral health. This may indirectly impact cardiovascular and infectious diseases, as periodontal health has been linked to reduced systemic inflammation and lower risks of heart disease [33,34].

Thus, this review aims to comprehensively explore the role of VIC in health maintenance and disease prevention. The present paper first sets a scientific foundation by analyzing VIC’s molecular structure, properties, and mechanisms of action. Then, the applications of VIC in preventing and treating various illnesses are thoroughly reviewed, highlighting this vitamin’s therapeutic potential. Further, VIC deficiency and supplementation are addressed, as they have important public health implications. This review also acknowledges the areas of ongoing debate and emphasizes the need for further research to solidify its role in clinical practice.

For this purpose, English-language publications (e.g., review articles, meta-analyses, preclinical studies, clinical trials, observational studies), mainly from the last seven years, are analyzed and discussed in this paper. These were searched in scientific databases such as Google Scholar, PubMed, Elsevier/Science Direct, Springer Link, Wiley Online Library, and Taylor & Francis online using various combinations between the following keywords: “Vitamin C”, “cardiovascular diseases”, “immune system”, “metabolic disorder”, “immune modulation”, “respiratory infections”, “wound healing”, “cancer treatment”, and “antioxidant effect”.

## 2. Biochemical Role of Vitamin C

### 2.1. Molecular Structure and Properties

**Figure 1:**
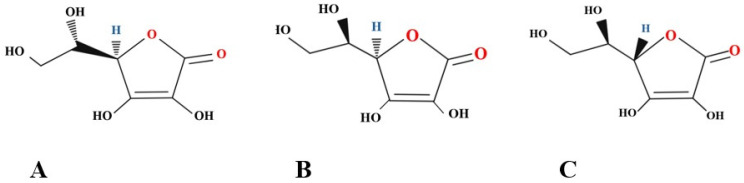
Isomers of vitamin C. (**A**) L-ascorbic acid, (**B**) erythorbic acid, and (**C**) D-ascorbic acid.

Vitamin C (C_6_H_8_O_6_), also known as ascorbic acid, is a simple carbohydrate with a low molecular mass and a structure similar to sugars, composed of six symmetrical carbon atoms. It is the aldono-1,4-lactone of a hexonic acid with an enediol group on carbons 2 and 3, capable of donating electrons. L-ascorbic acid isomers (Figure 1) erythorbic acid, known as D-araboascorbic acid, and D-ascorbic acid also have activity in the human body. Still, unlike L-ascorbic acid, they are more readily metabolized and eliminated from the body. Ascorbic acid is soluble in water, difficult to solubilize in alcohol, and impossible to solubilize in benzene, ether, or chloroform, so its chemical structure determines its physical and chemical properties. Although soluble in water, it is a weak, unstable organic acid that can be readily oxidized at high temperatures or in the presence of light, oxygen, or alkaline and high-humidity environments [24,35,36,37].

The use of derivatives, such as zinc ascorbate, calcium L-ascorbate (calcium ascorbate), sodium L-ascorbate (sodium ascorbate), calcium sodium ascorbyl monophosphate salt (sodium calcium ascorbyl phosphate), and 6-palmityl-l-ascorbic acid (ascorbyl palmitate), has contributed to the increased stability of ascorbic acid [24,35,36,37].

At a physiological pH of 7.4, L-ascorbic acid is found as the ascorbate monoanion that can remain in its reduced form (ascorbic acid—AA) or transform into its oxidized form (dehydroascorbic acid—DHA). Thus, AA can donate electrons and act as a reducing agent. It successively loses two electrons, the intermediate species composed of a single electron, or free radical, also called an ascorbate radical, which is stable and relatively non-reactive. The loss of the second electron leads to the appearance of its oxidized form, DHA. DHA can be rapidly degraded to 2,3-diketogulonic acid by reactive hydrolysis, the molecules thus losing their vitamin property. In aqueous environments, this degradation phenomenon is much more accentuated, with the oxidation of AA occurring rapidly. Also, metal ions (e.g., Fe^3+^) can lead to oxidation of AA and hydrolysis of DHA. However, dehydroascorbic acid can be reduced back to its ascorbic acid form in the body, this process being essential for its antioxidant activity [8,9,38,39].

Free radicals and ROS have a dual role in the body. Although they have beneficial effects, they can also be toxic compounds formed during metabolic processes and in response to exogenous stimuli. When they occur in excess and cannot be eliminated, their accumulation can lead to oxidative stress, and this process can lead to the development of chronic and degenerative diseases (e.g., cancer, cardiovascular disease, aging, cataracts). VIC can scavenge biological systems’ free radicals, protect lipid membranes and proteins from oxidative damage, and neutralize the reaction. Free radicals are unstable, so they require electrons to find stability. VIC is a reducing substance and an electron donor, so in the presence of free radicals, VIC donates high-energy electrons, neutralizing them. DHA is formed during this process, which can be recycled and reused, releasing more electrons. In cells, DHA is also reduced to generate AA, which protects mitochondria from oxidative damage by free radicals [40]. Thus, it has been observed that the antioxidant effect of VIC is closely related to its chemical properties. Due to its ability to donate electrons, it neutralizes free radicals and ROS. This property is also due to the redox ability that VIC has, being able to transition between its reduced form and its oxidized form [6,41,42].

Understanding the stability of VIC is essential to its possible use in biomedical applications. VIC is unstable in aqueous environments, at high pH, and in the presence of oxygen and metal ions, where its degradation is accelerated. A high pH leads to the decomposition of AA, and thus its efficiency decreases. High temperature also contributes to the breakdown of VIC. Thus, it has been observed that an increase of 10 °C leads to a doubling of the rate of VIC oxidation, while UV rays have been found to have the same effect. The presence of oxygen also accelerates the oxidation of VIC, contributing to the formation of DHA, which is more unstable and may degrade later. These issues are essential for the processing and storing of foods and supplements containing VIC [36,39,43,44].

### 2.2. Mechanisms of Action

VIC is vital in numerous and varied biological processes, particularly relevant as an enzyme cofactor. Thus, this role is closely related to dioxygenases involved in the synthesis of collagen and carnitine, in the transcription and regulation of gene translation, and the metabolization of tyrosine and monooxygenases involved in the synthesis of hormones. Regarding dioxygenases, VIC maintains the metals present (e.g., iron, copper) in their reduced state [24,45].

Enzymes that depend on VIC as a cofactor fall into two main categories: iron-dependent or 2-oxoglutarate-dependent dioxygenases. Iron-dependent dioxygenases catalyze hydroxylation reactions critical for collagen and carnitine synthesis, while 2-oxoglutarate-dependent dioxygenases (2-OGDDs) are involved in gene regulation and metabolic pathways. Enzymes of the 2-OGDDs family present non-heme iron bound to the enzyme with two histidine residues and an aspartic or glutamic acid through triple coordination. The other iron sites are occupied by water molecules, which are substituted by 2-oxoglutarate (2-OG) and oxygen (O_2_) during the catalytic reaction. Thus, the enzyme catalyzes the hydroxylation of the substrate, forming a superoxide radical that decarboxylates 2-OG, forming a ferryl intermediate, which helps to bring iron to its active state (Fe^2+^). VIC facilitates the reduction of iron (Fe^3+^) back to Fe^2+^, preventing an uncoupled cycle where 2-OG is oxidized to succinate and CO_2_ without hydroxylating the substrate. Without VIC, enzyme inactivation occurs rapidly but remains reversible [24,45].

Monooxygenases such as dopamine-β-hydroxylase and peptidyl-glycine α-amidating monooxygenase (PAM) also depend on VIC. Dopamine β-hydroxylase is present in catecholamine storage vesicles at the level of the nervous tissue but also in the adrenal glands. This dimer or tetramer enzyme, containing catalytic copper ions, relies on VIC to reduce Cu^2+^ ions at the active site and activate oxygen, enabling dopamine hydroxylation to norepinephrine. On the other hand, PAM is a bifunctional enzyme involved in the synthesis of hormones and neurotransmitters such as oxytocin, calcitonin, vasopressin, neuropeptide Y, GLP-1, and substance P, found in tissues such as the pituitary and adrenal glands. It exhibits two distinct catalytic domains, and its activation involves two steps: hydroxylation of the peptide precursor and the second involves amidation of the intermediate product. Both steps require copper, ascorbate, and oxygen. VIC is an important element in the function of both enzymes, and its deficiency can lead to decreased enzyme activity, which impairs the synthesis of important neurotransmitters and hormones [24,45].

In addition, VIC may also be a key factor for α-ketoglutarate dioxygenases (α-KGDD), maintaining and enhancing the activity of these enzymes. Thus, VIC is involved in epigenetic modifications of histones containing the Jumonji-C domain (JHDM) and the ten-eleven translocation (TET) family of DNA hydroxylases. It has been observed that VIC added to somatic cells can activate pluripotency genes, leading to epigenetic memory erasure in adult cells. Its action on α-KGDD may lead to the regulation of metabolism and DNA/RNA demethylation, inducing somatic cell reprogramming, and may even be important in DNA repair [46].

### 2.3. Antioxidant Function

Oxidative stress is an important toxic factor in the development of chronic diseases. VIC is a well-known antioxidant that can stop the production of ROS and reactive nitrogen species (RNS) through two pathways. The first direct pathway is the inhibition of nitrogen oxides, preventing the formation of radicals such as hydroxyl, peroxyl, and nitroxide. On the other hand, VIC may be a substrate involved in ascorbate peroxidation, converting hydrogen peroxide to water [42,47]. VIC may also boost the activity of vitamin E (α-tocopherol), enhancing its antioxidant effect by controlling lipid peroxidation in cell membranes and nuclear material. Specifically, the generated α-tocopheroxyl radical reacts with AA to regenerate α-tocopherol, while AA is oxidized to ascorbyl radical [6,41]. As previously mentioned, the antioxidant activity of VIC is closely related to its chemical properties. Its ability to donate electrons to neutralize free radicals and ROS is due to its property of going from its reduced form to its oxidized form and vice versa, which contributes to this antioxidant activity [42].

Due to its antioxidant properties, vitamin C is being studied to prevent diseases associated with oxidative stress. Several studies have investigated the potential of VIC for cardiovascular diseases, including hypertension [48], atherosclerosis, and coagulation abnormalities [49,50,51]. It was also found that VIC exerts its antioxidant role not only by its action on ROS but also by redox regulation and enhancement of the activity of other cellular radical scavengers [48]. However, analysis of several studies has shown that the association of VIC with the risk of coronary heart disease is controversial. The source of VIC is an important factor, and its levels in the blood must be constantly monitored to determine the impact of VIC on the prevention of cardiovascular disease [52].

Furthermore, oxidative stress may also be associated with neurodegenerative diseases, and research has found high concentrations of VIC in the brain. Thus, VIC may also be involved as a neuroprotective. Firstly, VIC is involved in catecholamine synthesis and has a regulatory effect, modulating enzyme hydroxylation. It has also been observed in mouse models that VIC can improve cognitive activity in schizophrenia and Alzheimer’s disease. VIC has also been studied as an antidepressant, contributing to the activity of 5-HT 1A and 5-HT 2A/2C receptors. However, further studies are needed to concretely determine the mechanisms of action of VIC on the nervous system and its use in the treatment of neurodegenerative diseases [53].

Numerous studies have investigated the effect of VIC concentration on cancer cells [54]. Thus, some in vitro experiments have used concentrations of 20 mM VIC and have been shown to selectively kill cancer cells without affecting healthy cells. The mechanism underlying this effect (cancer cell killing) was associated with forming hydrogen peroxide and forming VIC radicals as intermediates. Also, the doses of VIC administered in the mouse model led to a halt in tumor progression, promoting a pro-oxidant effect. VIC also potentiated the effect of anti-tumor drugs, such as gemcitabine, against pancreatic cancer cell lines, as well as drugs used in ovarian cancer. It has also potentiated the effect of radiotherapy and managed the side effects of this procedure [55,56].

### 2.4. Role in Collagen Synthesis

Collagen (Col) is an essential protein for the human body. It has a triple-helix structure with three identical or different α-chains, which mimics and can maintain the interaction between cells and the matrix [57]. It can be classified as microfibrillar, short-chain, transmembrane, anchoring, basement membrane, fibril-associated collagen with interrupted triple-helix (FACIT), and fibrillar collagen [58]. It is found in skin, organ capsules, bones, tendons, cornea, muscle fascia, and blood vessels. It plays an important role in the body, maintaining mechanical strength, elasticity, and support of the tissues, being a major component in the extracellular matrix of cells (ECM). Besides supporting cells, collagen is also involved in cellular activity and behavior, such as cell proliferation, migration, and differentiation [59].

There are about 30 types of collagens. Type I collagen is the most prevalent collagen in the human body, found in connective tissues, tendons, ligaments, bones, and skin. It is composed of two identical α1 (I) chains and one α2 (II) chain in each structural domain of bone; collagen fibers lead to bone mineralization, either directly or in association with other proteins responsible for mineralization. Collagen is also involved in other physiological processes, such as homeostasis and angiogenesis, and is also present in pathologies such as cancer, fibrosis, or atherosclerosis [59,60].

VIC is an essential cofactor in collagen synthesis and acts on the enzyme’s prolyl hydroxylase and lysyl hydroxylase in this process. These enzymes are responsible for cross-linking collagen molecules by hydroxylating proline and lysine residues and help stabilize collagen’s triple-helix structure. This process leads to the formation of mature and solid collagen fibers. Furthermore, VIC is involved in gene expression for collagen types I and III and may also protect collagen from oxidative stress, which could hinder various processes such as wound healing. Hydroxylation of proline residues occurs at the γ-C atom and sometimes at the β-C atom, whereas lysine occurs at the δ-C atom. These reactions have as catalysts VIC-dependent dioxygenase enzymes such as prolyl 4-hydroxylase, prolyl 3-hydroxylase, and lysyl 5-hydroxylase [26,41,61]. Also, not only is it involved in collagen synthesis, but VIC may also be active in collagen gene expression. It has been observed that VIC can increase the messenger RNA (mRNA) levels for type I and III collagen in fibroblasts, leading to mRNA stability and, thus, more efficient collagen production. It was also found that VIC can inhibit the synthesis of certain types of collagen (types V and VI), allowing for the body to select the type of collagen required for its needs. VIC may interact with growth factors (e.g., epidermal growth factor (EGF) and fibroblast growth factor (FGF)), contributing to the regeneration of injured tissues by collagen synthesis [62].

## 3. Pharmacokinetics and Supplementation

### 3.1. Absorption, Distribution, Metabolism, and Excretion (ADME)

The pharmacokinetics of VIC is a complex process that requires maintaining the body’s homeostasis. It involves the following steps: absorption, distribution, metabolism, and excretion. It is important to understand the kinetics of VIC so that it can be properly administered to patients according to individual needs and specific conditions. Genetic factors, lifestyle, and patient comorbidities may influence this process. Also, the pharmacokinetics of VIC may be influenced by the dose administered, making it difficult to obtain data on its therapeutic effect. Studies have shown that VIC is subject to zero-order to first-order pharmacokinetics, making it difficult to predict its possible effects, especially when high doses are administered [24,63,64].

**Figure 2:**
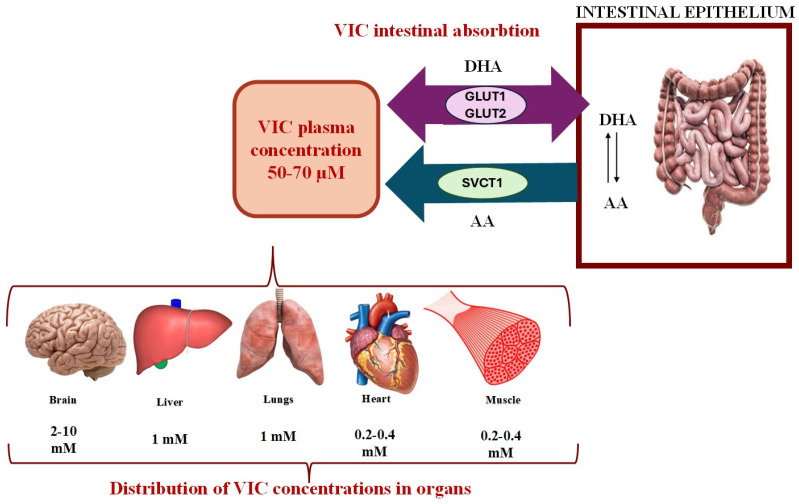
VIC absorption process and concentration distribution in organs. Created based on information from [64,65].

Absorption of VIC (Figure 2) occurs mainly in the intestine, specifically in the distal ileum, via sodium-dependent transporters (SVCT1 and SVCT2). This process is saturable and dependent on intestinal pH and represents the decrease in absorption capacity at high concentrations of VIC. At low doses, VIC is absorbed in a high proportion. VIC can also be absorbed by passive diffusion, especially at low pH, where VIC is non-ionized. In the small intestine, VIC exists as ascorbate (predominant) and DHA, which is reduced to the ascorbate form in cells. DHA is absorbed through glucose transporters (GLUT1 and GLUT2) [24,63,64].

VIC is distributed differently in the body (Figure 2), with its concentrations varying in different organs. It can accumulate in the brain and adrenal glands (10 mM), where there are the highest concentrations of VIC, while in the muscle and myocardium, concentrations are lower (0.2 mM). VIC plasma levels are 40–65 µM in healthy adults, reaching a plateau of 200–400 mg/day. Cells contain concentrations 5 mM higher than the plasma level (except erythrocytes), while neuronal cells and endocrine cells have VIC levels of 10 mM, as VIC is involved in synthesizing neurotransmitters and hormones [24,63,64].

Excretion of VIC occurs mainly in the kidney. The SVCT1 transporter contributes to the reabsorption of VIC from the primary urine; thus, its excretion depends on the saturation of this process. VIC in low doses is not eliminated, whereas doses ≥ 500 mg lead to almost complete elimination of VIC in the urine [24,63,64].

### 3.2. Recommended Dietary Allowances

VIC is an important nutrient in the human diet, and an adequate intake of it can be obtained by eating fresh fruits and vegetables. Nutritionists recommend five servings of fruits and vegetables per day, which could provide about 200 mg of VIC [66]. In a study by the US National Health and Nutrition Examination Survey (NHANES) conducted between 2017 and 2018, only 8% of the adult population met this dietary threshold, with patients having an average VIC concentration of 66 μmol/L. Also, the bioavailability of VIC in whole foods is 76%, compared to 70–90% when taking VIC-based supplements [67,68,69].

Thus, the recommended VIC dose was 90 mg/day for adult males, while male adolescents need 75 mg/day. Generally, women need 75 mg/day; however, for pregnant and breastfeeding women, a higher dose is indicated, while 65 mg/day is recommended for teenage females. In children, the daily dose of VIC is according to their age. For people who smoke, the dose of VIC should be supplemented by 35 mg due to the deficient absorption of VIC caused by this habit [66]. For an at-glance perspective over these quantities, the dietary recommended allowances for VIC are summarized in Table 1.

To combat VIC deficiency, there is no clear protocol regarding its dosage in cases of major deficiency. In cases of deficiency in newborns (from birth to 28 days), the recommendation is 50 mg/day orally or intravenously. Also, for premature babies, the recommended dose is 25 mg/day orally or intravenously. Children weighing less than 10 kg are recommended 125 mg/day orally or 75 mg/day intravenously. Those over 10 kg up to 40 kg may receive 500 mg/day given twice or 75 mg/day intravenously. Those over 40 kg should receive 1000 mg daily orally or 500 mg/day intravenously. These dosages should be administered according to the needs of the deficient patients, and this should be stable based on blood levels of VIC and re-evaluation of these levels 2–4 weeks after starting supplementation [67,68,69].

### 3.3. High-Dose Vitamin C: Safety and Efficacy

In addition to the beneficial effects that VIC can have in high concentrations, there are also consequences of administering it in this way. It has been found that high levels of VIC may be associated with the development of kidney stones, as one of the metabolites of VIC is oxalate. The development of acute oxalate nephropathy has also been observed in patients after intravenous administration of high concentrations of VIC to patients with chronic kidney disease. High intravenous concentrations of VIC may also produce adverse effects in some categories of patients. In patients with glucose-6-phosphate dehydrogenase (G6PD) deficiency, intravascular hemolysis may be produced, and oral administration may have a similar effect on patients with paroxysmal nocturnal hemoglobinuria. On the other hand, following intravenous administration of VIC, some patients have reported adverse effects such as dizziness and nausea, which improved after the consumption of food and water. Vomiting, thirst, dry mouth and skin, increased urinary flow, diarrhea, headache, chills, imbalance, edema in the legs, hypertension, insomnia, loss of appetite, fatigue, etc., were also reported as adverse effects. In laboratory tests, administration of high concentrations of intravenous VIC was also associated with hypernatremia, hypokalemia, hypercalcemia, and a low hemoglobin concentration [67,68,69,70].

To avoid such side effects, the VIC supplementation should not exceed the tolerable upper intake levels (Table 2).

## 4. Vitamin C in Disease Prevention

### 4.1. Cardiovascular Health

Cardiovascular diseases (CVDs) are disorders associated with the circulatory system (heart and blood vessels) and are among the most prevalent conditions in the world’s developed countries, with increased mortality rates. CVD can remain hidden and undiagnosed for a long time. The conditions that lead to CVD are high blood pressure (hypertension), coronary artery disease (CAD), and atherosclerosis. Atherosclerosis is characterized by the thickening and hardening of the artery walls due to elevated plasma cholesterol levels, while CAD is characterized by narrowing or blockage of the coronary arteries. In CAD, fatty plaques form and grow inside the blood vessels, causing severe inflammation. The consequences of CAD include heart attack, stroke, ischemia, or even death. Arterial hypertension (AH) is the most common and frequently encountered cardiovascular disease and is more dangerous because it has little or no symptomatology, but it can cause heart attack, stroke, kidney failure, and peripheral vascular disease [63,64,65].

The vascular endothelium is essential in the functions of the circulatory system by maintaining homeostasis, while its dysfunction is a key factor in the development of both atherosclerosis and CAD. In atherosclerosis, arterial regions with turbulent blood flow, such as branch points, are particularly prone to lesions due to low shear stress and endothelial damage. The endothelial cells constantly produce nitric oxide, which regulates anti-inflammatory and anti-thrombotic processes. However, during inflammation, these processes are impaired, and lipoprotein oxidation is intense and allows for their accumulation in the subendothelial space. Nitric oxide depletion and ROS generation promote vasoconstriction and procoagulation, leading to the development and degeneration of atherosclerosis. In atherosclerosis, inflammation plays an important role, since oxidized lipids activate macrophages and T lymphocytes, which lead to the secretion of inflammatory cytokines (TNF-alpha, IL-1, IL-6) and the appearance of ROS. These processes facilitate the appearance of more leukocytes, which modify lipoproteins and transform them into atherosclerotic plaques and chronic inflammation. In the case of CAD, instead of nitric oxide, superoxides occur, leading to oxidative stress, which damages the vasculature and leads to atherosclerotic plaques and chronic inflammation [63].

Over the years, researchers have focused on finding ways to effectively prevent, treat, and control CVD. VIC has become a substance of interest because of its antioxidant properties, which could decrease CVD risk. People with a high concentration of VIC had a low risk of stroke, while people with a normal or deficient concentration of VIC had twice the risk. Lack of VIC may favor the development of oxidative stress and, thus, atherosclerosis [65,66].

Studies conducted between 1990 and 2000 have had conflicting results. Some showed that daily intake of VIC could not be associated with a lower risk of developing CAD, while others suggested a decreased risk of developing CAD in the patients involved in the study. Meta-analyses and additional studies concluded that VIC intake is inversely associated with CAD risk. Some studies showed benefits, while others did not, with no evidence of cardiovascular events [52,67,68,69,70,71,72,73,74]. A meta-analysis conducted in 2017 [75] showed that vitamin C supplementation could reduce cardiovascular events and mortality, but data are insufficient. A recent Mendelian randomization (MR) study was performed by Zhu et al. [76] in 2021 to examine the association of genetically predicted blood concentration of VIC with CVD events such as CAD, atrial fibrillation, ischemic stroke, heart failure, and CVD risk factors such as serum lipids (high-density lipoprotein (HDL), low-density lipoprotein (LDL), triglycerides), blood pressure, and body composition. The study used two sample MR analyses, concluding that VIC’s genetically predicted blood concentrations do not influence the potential to produce major CVD, suggesting that VIC supplementation may not help prevent CVD. Chen et al. [77], in an MR study realized in 2021, wanted to show the correlation between the levels of VIC plasma concentration and the risk of CVD. They applied two-sample MR to highlight the causality of VIC with numerous CVD outcomes such as CAD, myocardial infarction (MI), atrial fibrillation (AF), heart failure (HF), stroke, and ischemic strokes and their subtypes. The study concluded that VIC plasmatic concentrations were associated with a lower risk of cardioembolic stroke and sufficient VIC intake might decrease the risk of this condition. Still, it has no effect in decreasing the risk of CAD, MI, AF, HF, or IS.

Several studies have also investigated VIC supplementation in hypertensive (but not in normotensive) patients, concluding that it reduced AH. VIC infusions also improved blood flow and reduced the susceptibility of LDL to oxidation [72,73,74,75,78,79,80,81,82,83,84,85,86,87,88,89,90]. A study from 2020, realized by Wang et al. [91], explored the potential of VIC to enhance low-density lipoprotein receptors and proprotein convertase subtilisin/kexin 9 (PCSK9). The in vitro experimental study was conducted on human liver cell lines and primary hepatocytes from animal models. Thus, the researchers demonstrated that VIC could inhibit PCSK9 expression at the protein and transcriptional level via the transcription factor FoxO3a, which blocks PCSK9 activation by proteins such as HNF1a. It was also observed that VIC can increase LDL receptor (LDLR) expression by reducing its PCSK9-mediated degradation. VIC also improves the stability of LDLR messenger RNA and activates SREBP2, another transcription factor. This may demonstrate that VIC supplementation may improve lipid profiles in VIC-deficient individuals and reduce CVD risks by lowering LDL levels and improving cholesterol metabolism. On the other hand, a study conducted by Boonthongkaew et al. [92] aimed to evaluate the effects on arterial blood pressure and oxidative stress following low-intensity exercise in patients with poorly controlled type 2 diabetes. The study was a randomized, controlled, double-blind, placebo-controlled, cross-over study in 24 patients (20 women, 4 men) with type 2 diabetes. Thus, patients were orally administered 1000 mg/day VIC for 6 weeks, their blood pressure was measured before and after exercise, and their oxidative stress markers (malondialdehyde-MDA, F2-isoprostanes) and nitric oxide (NO) levels were also monitored. Blood pressure and blood samples were taken before, immediately after, and 60 min after exercise. The results show that blood pressure fell in patients given VIC compared with placebo. The same result was also observed after 60 min. Also, plasma levels of MDA and F2 were significantly lower in the group receiving VIC, while NO levels increased significantly before and after exercise. Thus, the researchers conclude that oral supplementation with 1000 mg/day of VIC led to decreased plasma lipid peroxidation biomarkers of lipid peroxidation as measured by MDA and F2-IsoPs concentrations and increased NO concentration, which appears responsible for the hypotensive effect. Another study conducted in 2018 had similar results regarding the potential of VIC to lower blood pressure and oxidative stress levels in patients with type 2 diabetes [93].

Vasoplegic syndrome is another condition in which VIC may represent an alternative route for prevention and treatment. The vasoplegic syndrome is defined as low systemic vascular resistance, normal or high cardiac output, and hypotension that is unresponsive to treatment with vasopressor agents. This condition may be considered when perioperative refractory hypotension develops after cardiovascular or transplantation surgery. In this regard, VIC can reduce inflammation and enhance microcirculation. Also, VIC can reduce oxidative stress. It can increase susceptibility to catecholamines, like corticosteroids; restore the systemic vascular tone; and reduce the vasopressor need [94]. In two double-blinded randomized clinical trials conducted in two intensive care units in Perth, Australia [95], VIC was administrated intravenously in patients with vasoplegic shock. In this way, the researchers wanted to highlight if intravenous VIC can reduce vasopressor requirements. For this purpose, 71 patients were selected, 35 patients received the treatment with VIC (intervention group), and 36 patients represented the placebo group. The patients were randomly selected to receive VIC or placebo based on a computer randomization system with 1:1 allocation. The patients in the intervention group received 1.5 g sodium ascorbate in 100 mL normal saline every 6 h, for 15 min, over 5 days. The treatment was administrated till the vasoactive therapy was stopped when the mean arterial pressure was achieved (>65 mm Hg). Patients on dialysis received a higher concentration of sodium ascorbate (3 g) due to high clearance due to dialysis, under the same conditions as patients without renal replacement. The primary outcome was the duration of vasopressor usage in hours. Secondary outcomes were intensive care unit (ICU) and hospital length of stay, as well as 28-day ICU and hospital mortality. However, patients who were given at least a moderate dose of vasopressors in the ICU showed that intravenous vitamin C administration, compared with placebo, did not significantly reduce vasopressor duration.

Two meta-analyses have demonstrated improved endothelial function in patients with atherosclerosis and diabetes, improving arterial stiffness. Other studies have shown that high plasma concentrations of VIC contribute to stroke. However, some studies contradict this association. One meta-analysis showed that VIC, vitamin E, and beta-carotene supplementation had no significant effect on stroke risk [72,73,74,75,78,79,80,81,82,83,84,85,86,87,88,89,90]. Thus, it can be concluded that there are currently not enough recent studies and insufficient data to determine the potential of VIC to reduce the risk of CVD. However, the studies presented above may be a key point for future research.

In this regard, more studies need to be conducted considering additional criteria, such as the concentration of VIC in the blood of patients before starting the study, as well as the amount of VIC administered during the study and the diet of patients. Studies should also be carried out on several patients to establish a therapeutic dose, mode of administration, optimal targets, and even the need to administer other antioxidants simultaneously with VIC [52].

### 4.2. Cancer Prevention

Cancer remains one of the three leading causes of death worldwide, alongside infectious and cardiovascular diseases. The disease is characterized by abnormal cell growth that has undergone a lot of damage and changes in the cell cycle and metabolism. A critical aspect of this disease is finding optimal treatment that prevents cancer cells from spreading throughout the patient’s body and metastasizing. This should involve destroying the malignant cells without causing adverse effects or worsening patients’ health [96,97]. Due to the very high incidence of new cases, 19.3 million cases, and high mortality, with almost 10 million deaths reported in 2020, the need for efficient treatment is increasing [96,98].

VIC can be used as a chemopreventive agent for various forms of cancer. Its antioxidant effects and anti-inflammatory and anti-metastatic potential play an essential role in this process, improving intracellular communication. As previously mentioned, VIC decreases oxidative stress by neutralizing free radicals, preventing DNA degradation, and inhibiting the formation of nitrosamines (i.e., substances with carcinogenic potential). VIC also reduces the inflammation associated with carcinogenesis by reducing the occurrence of nitric oxide and the activation of nuclear factor kappa B (NF-κB). It can inhibit the activity of metalloproteinases (MMPs), which can lead to the degradation of CME and the development of metastases [99,100].

It has been observed that VIC cannot be associated with a lower risk of developing cancer, especially breast, endometrial, bladder, esophagus, lung, stomach, prostate, and kidney cancers. A Mendelian randomization study [101] collected data on several types of cancer, which have been mentioned above. Unfortunately, the study did not find conclusive evidence on the concentration of VIC and the incidence of the cancers examined. However, daily dietary intake of VIC was associated with a lower risk of lung cancer but not other cancers. In another meta-analysis [102], dietary intake of VIC from food was associated with an 18% decreased chance of developing lung cancer, but the supplements did not show any real benefit. At the same time, the antioxidant effect of VIC has been observed in preventing ROS and DNA damage. However, at high concentrations, VIC has a pro-oxidant effect, and the association with certain factors, such as smoking, could create a damaging effect. It was observed that an intake of VIC from fruits and vegetables may lead to the prevention of lung cancer, while supplements do not provide such benefits and are associated with a harmful effect [101,102,103].

### 4.3. Neuroprotective Effects

Worldwide, neurodegenerative diseases have been on the rise substantially, and it is now predicted that around 130 million people will be affected by dementia by 2050. Dementia is a neurodegenerative disease that leads to disability, institutionalization, and mortality, while Alzheimer’s disease accounts for 60–70% of dementia patients. Alzheimer’s disease is initially characterized by episodic memory loss, followed by deterioration of cognitive activity, such as difficulties with speech, executive and visuospatial activity, and eventually dementia. Parkinson’s disease is the second most common neurodegenerative disorder, which is characterized by bradykinesia, muscle rigidity, tremor, and impaired locomotor function and posture in individuals. This is due to the loss of dopamine-producing neurons [104].

The brain is prone to oxidative damage because it uses reactive species to transmit signals. It consists of about 86 billion neurons and about 300 billion glial cells, which are the main consumers of basal oxygen. At the same time, the human brain exhibits high oxidative metabolic activity, and thus, there is an intense production of ROS, which can damage neuronal cells that are not replicable. However, there are protective mechanisms, such as a cellular antioxidant system that prevents tissue damage, consisting of exogenous and endogenous antioxidants, which are designed to reduce various chemicals. Free radicals and other reactive molecules can disrupt cellular energy metabolism, causing oxidative stress and leading to a major imbalance between pro-oxidants and antioxidants. These radicals can further damage lipids, proteins, and nucleic acids, which can cause structural and functional changes. The brain has a high lipid content, which is the main target for oxidative damage. This can lead to neurodegenerative diseases such as Alzheimer’s and Parkinson’s. Although neurons have mechanisms against oxidative stress, these are insufficient and make them vulnerable to redox reactions [105].

In certain brain regions, VIC reaches high concentrations needed to prevent oxidative stress and maintain redox balance. VIC is essential in redox reactions catalyzed by enzymes such as dopamine β-hydroxylase, which converts dopamine to norepinephrine, and other enzymes involved in synthesizing neuropeptides and arachidonic acid. VIC also contributes to myelination and the differentiation of neuronal precursors, playing a role in the development and maturation of the central nervous system. Deficiencies of VIC can lead to neuropsychiatric disorders, and many studies are trying to highlight its effect as an adjuvant therapy in this regard [106,107]. VIC may act as a neuroprotectant due to its antioxidant properties that allow for it to attenuate oxidative stress generated by various neurotoxins. In some cases, it is considered that VIC may also play a role in recovery after brain trauma and an effect against brain aging [106].

VIC is an important element in the optimal functioning of the central nervous system (CNS), where it acts as a powerful antioxidant. It plays a role in the differentiation and maturation of neurons, in the synthesis of catecholamines and neurotransmitters, and also contributes to the synthesis of collagen and elastin, which are essential for the structure of the CNS [108,109,110]. Treatment with divalent cations (e.g., iron and copper) leads to the formation of α-synuclein oligomers in the presence of DOPAL (dopamine aldehyde or 3,4-Dihydroxyphenylacetaldehyde), which is a neurotoxic product of dopamine metabolization. VIC can prevent this process by inhibiting DOPAL oxidation or interaction with divalent cations. In an experimental model of Parkinson’s disease (PD), it was observed that VIC favored the activity of antioxidant enzymes, preventing the onset of oxidative stress and the loss of dopaminergic neurons. Also, VIC acts as an activator for ten-eleven-translocation 1–3 (Tet1–3) and Jumonji C domain-containing histone demethylases (Jmjds), which have direct action in the demethylation of histone lysine residues and in the differentiation of dopaminergic neurons [111]. Unevenly distributed concentrations of VIC in the CNS may be a key factor in increasing susceptibility to oxidative stress, especially when dopamine is metabolized, which generates ROS and leads to specific protein accumulation in PD. Studies are attempting to associate VIC with this disease, and it has been found that VIC deficiency is a hallmark of PD. Some studies have shown that VIC-based supplements have been effective in ameliorating the effects of oxidative damage in experimental models, while a meta-analysis has emphasized that VIC from supplements has no effect in preventing PD. One explanation for this phenomenon may be attributable to the solubility of VIC, which must be transported across the blood–brain barrier in an active mode to reach the CNS. However, it has been observed that in patients with PD receiving levodopa, VIC contributes to an improved effect of this drug while also reducing the dosage and adverse effects [108,109,110].

VIC also plays an important role in the pathophysiology of Alzheimer’s disease since its antioxidant and neuroprotective properties are exerted on ROS, but also on amyloid plaques and neurofibrillary tangles characteristic of this disease. It is released by glial cells into the synaptic clefts and contributes to replenishing antioxidant enzymes such as glutathione and catalase, reducing oxidative stress and inflammation. VIC can also destabilize amyloid fibers, thus being a protective factor against fiber cytotoxicity. Studies in experimental models have suggested that VIC improves cognitive function and reduces the appearance of ROS, but in human subjects, this is not the case. However, an increase in the efficacy of VIC-associated treatments for Alzheimer’s and a reduction in the toxic effects of amyloid plaques was observed. It was also observed that low levels of VIC in plasma and cerebrospinal fluid in patients diagnosed with Alzheimer’s disease are closely related to advanced stages of the disease and correlated with reduced antioxidant activity [112,113].

### 4.4. Chronic Diseases Prevention

It is well known that oxidative stress contributes to the development of chronic diseases, such as diabetes mellitus, cardiovascular diseases, neurological disorders, psychiatric diseases, lung diseases, and many others. Thus, the effect of both antioxidant enzymes and small-molecule antioxidants (e.g., vitamin C) against oxidative stress and how their action prevents the development of chronic diseases is being studied [114].

For example, metabolic syndrome is characterized by several metabolic abnormalities such as large abdominal circumference, high blood pressure, high blood glucose, high triglyceride levels, and low HDL cholesterol levels, and is associated with an increased risk of CVD, type 2 diabetes, and stroke. In this regard, a meta-analysis has been conducted to demonstrate the role of VIC in managing this metabolic syndrome, which can lead to coronary heart disease. In human studies, it was observed that patients suffering from metabolic syndrome have a low concentration of VIC in the blood. It is known that oxidative stress and inflammation are correlated and that they lead to the negative evolution of metabolic syndrome. Through its antioxidant and anti-inflammatory properties, VIC contributes to alleviating these symptoms and complications associated with metabolic syndrome. Thus, in experimental animal models, the administration of VIC significantly contributed to weight loss and glycemia levels, stabilization of blood pressure, and an improved lipid profile by decreasing LDL and increasing HDL, and these effects were further observed in human studies. In this case, VIC acted as a reducing agent that neutralized free radicals, and the regulation of the inflammatory response was associated with a reduction in levels of inflammatory proteins (e.g., CPR-C-reactive protein), stimulating the immune system to fight against inflammation. VIC also stimulated carnitine synthesis, reducing body fat accumulation and influencing glycolysis by modulating hypoxic signaling [115]. Another study [116] conducted on the effects of natural antioxidants, including VIC, has shown a huge potential in reducing oxidative stress and improving insulin sensitivity in patients with diabetes mellitus, but also a potential in preventing the onset of this disease. As mentioned above, VIC helps lower blood pressure by improving blood flow, reducing LDL in healthy patients and patients suffering from other conditions such as diabetes mellitus, and preventing stroke [72,73,74,75,78,79,80,81,82,83,84,85,86,87,88,89,90].

Osteoarthritis is also considered a chronic disease that can lead to a deterioration of joint functions, such as stiffness and loss of motor skills. VIC has been studied as a potential treatment for this condition due to its antioxidant potential and its action in collagen synthesis as a cofactor. Here again, studies are contradictory regarding the action of VIC in combating this condition [117,118]. It has been shown to have a protective effect against cartilage degeneration, and in high concentrations, VIC may worsen osteoarthritis in experimental models [118]. In humans, it has been observed that VIC may prevent the onset of osteoarthritis, but also vice versa, with high concentrations of VIC being associated with an increased risk of developing osteoarthritis [117] and VIC did not prevent damage to cartilage structure [117,118].

## 5. Therapeutic Applications of Vitamin C

### 5.1. Infectious Diseases

**Figure 3:**
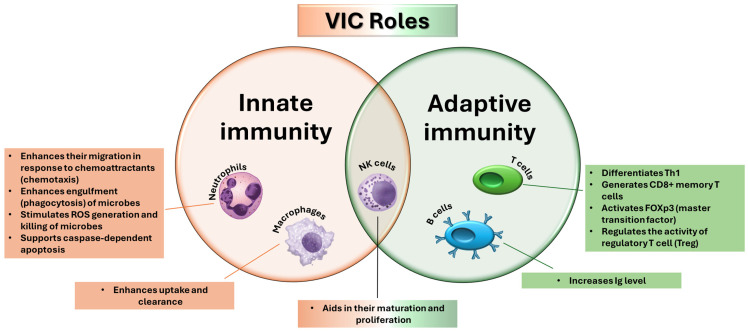
Schematic representation of VIC roles in innate and adaptive immunity. Created based on information from [121,122,123].

The immune system is the body’s defense against pathogenic agents (e.g., bacteria, viruses, fungi, parasites), cancer cells, and toxins. Its function is fulfilled by different organs, tissues, proteins, and chemicals and is simply classified into two defense categories: innate immunity and adaptive immunity. The first line of defense against invasive infection is innate immunity. The host utilizes this antigen-independent (non-specific) defense mechanism as soon as possible or within a few hours of contact with an antigen. Because the innate immune response has no immunological memory, it cannot recognize or “memorize” the same pathogen if the organism is re-exposed to it. On the contrary, adaptive immunity involves a lag period between exposure to the antigen and the maximal response because it is antigen-specific and antigen-dependent. Memory capacity allows for the host to develop a faster and more effective immune response to future re-exposure. Adaptive immunity and innate immunity are complementary defense processes for the body; deficiencies in either system make the organism vulnerable or cause inappropriate responses [119,120,121]. VIC is essential in enhancing the immune system by supporting various cellular functions of innate and adaptive immune systems, as summarized in Figure 3.

Vitamin C plays a crucial role in innate and adaptive immunity, influencing the epithelial barrier, supporting phagocytosis, and enhancing lymphocyte differentiation. These functions make it vital in preventing and treating infections [121,122]. Infectious diseases are caused by microorganisms that can be easily transmitted from one person to another, which can arise or re-emerge under specific conditions or situations. They can be new infections resulting from changes in pathogenic organisms or known infections that spread and re-emerge due to pathogens’ resistance to treatment [124].

In this regard, vitamin C has long been studied, and its deficiency has been associated with diseases such as pneumonia. Subsequently, numerous other studies have investigated it to fight other types of infections. It has been observed that VIC can prevent and help fight infections caused by viruses, bacteria, or protozoa. Infections cause oxidative stress in the human body, and any effect of VIC may be related to this. Infections lead to the activation of phagocytes, which cause ROS and are involved in processes that aim to inactivate viruses and kill bacteria. However, ROS can hurt host cells; VIC comes in with its antioxidant property, protecting cells from ROS. VIC is absorbed by phagocytes and is subsequently converted into DHA. Lymphocytes also need high concentrations of VIC (10–100 > plasma concentration) to help their proliferation and immune cell differentiation. VIC also contributes to regulating macrophage functions and improves innate immunity by inhibiting monocyte apoptosis and reducing the secretion of pro-inflammatory cytokines (e.g., IL6 and TNF-α). For neutrophils, VIC acts as a protector against the oxidation of cell membranes. Thus, VIC contributes to improved motility and phagocytosis of pathogens. In this sense, plasma VIC concentration decreases during infections, and supplementation may be a therapeutic strategy for treating infections [123,125,126].

It has also been observed that VIC exhibits antimicrobial properties, inhibiting the growth of several pathogens, such as *Pseudomonas aeruginosa*, *Staphylococcus aureus*, and *Escherichia coli*, and at high concentrations, can also inhibit biofilm formation. VIC can also inhibit the replication of viruses like herpes simplex and rabies viruses and has antiparasitic effects, reducing the number of parasites in *Trypanosoma cruzi* and *Plasmodium* infections. In addition, vitamin C can inhibit the morphogenesis of some fungi, such as *Candida albicans* [123,127].

By its antioxidant effect, VIC can inhibit the oxidation of proteins, lipids, carbohydrates, and nucleic acids and affects the regulation of immune function by acting as a cofactor for enzymes involved in gene synthesis and expression. VIC has been observed to influence the integrity of the epithelial barrier, activate NK cells, and help differentiate CD4+ T cells into T helper (Th1) cells that produce interferon-gamma (IFN-γ). VIC can be absorbed in leukocytes in high concentrations (50–100 times higher than plasma concentrations), providing an antioxidant effect [55]. VIC influences leukocytes, and supplementation leads to enhanced mediated response (e.g., lymphocyte proliferation, delayed-type hypersensitivity response, antimicrobial activity). It was observed that 1 g/day of VIC for 28 days increased T lymphocyte proliferation and the production of cytokines and immunoglobulins to enhance the immune response against infection. Phagocytic cells (e.g., neutrophils and macrophages) show a high concentration of VIC, which stimulates their migration to the site of infection and enhances phagocytosis, oxidant generation, and pathogen killing. However, VIC also prevents excessive action of the immune system and reduces tissue damage by increasing neutrophil apoptosis and their elimination by macrophages. This process prevents necrosis [119,120,121,128].

VIC may also influence the production of cytokines, which are secreted by immune cells in infection and inflammation and are responsible for regulating both humoral and cellular immune responses, eliciting pro-inflammatory or anti-inflammatory responses. Cytokines are categorized into chemokines, interferons (IFN), interleukins (IL), lymphokines, and TNF. In vitro studies have shown that VIC has the potential to decrease the secretion of the pro-inflammatory cytokines TNF-α and IFN-γ while considerably increasing the production of IL-10. In patients with pneumonia, VIC also increased TNF-α and IL-6, enhanced antiviral IFN generation in virus-infected fibroblasts, and normalized cytokine generation depending on the inflammatory stimulus and cell type [121].

### 5.2. Respiratory Illnesses

The common cold is one of the most common upper respiratory tract viral infections, with mild symptoms associated with a sore throat, cough, fever, fatigue, and muscle aches. It lasts from a few days to a maximum of three weeks. The rhinovirus is the main pathogen responsible for the common cold, present in 30–50% of cases [4,123,126,129,130].

In the 1970s, Nobel Prize-winning researcher Linus Pauling proposed using VIC to prevent and relieve the symptoms of colds. A review of placebo-controlled studies [131] concluded that taking 200 mg doses of VIC did not decrease the incidence of colds in the general population but significantly affected people under physical exertion (e.g., athletes). It was also found that the administration of VIC at the onset of colds showed no clear benefit, but regular administration reduced the duration of infection by 8% in adults and 14% in children. Placebo-controlled studies have shown that VIC can reduce the severity and duration of symptoms, with some reporting a reduction of as little as 5% in some cases and others reporting a reduction of 50%. The benefits of VIC against colds have been found in people who have had a low intake of VIC and in adults who are in constant contact with children [4,123,126,129,130]. Also, a study carried out in 2019 by Kim et al. [132] aimed to show that oral supplementation with VIC can reduce the chance of developing a common cold in army recruits from the Republic of Korea. The study involved 1444 participants who were divided into two groups. The first group (695 people) received 6000 mg/day of VIC, while the second group (749) received a placebo for 30 days. The study thus demonstrated that taking VIC can reduce the occurrence of common colds in recruits who are undergoing basic military training.

Pneumonia is a severe lower respiratory tract infection caused by pathogens such as *Streptococcus pneumoniae* and *Haemophilus influenzae*, which manifests as cough, fever, muscle aches, dyspnea, sputum production, and chest pain. Untreated pneumonia can lead to acute respiratory distress syndrome (ARDS), which manifests as hypoxia and pulmonary edema [4,130,133,134]. It was observed that VIC reduced respiratory symptoms in people with severe pneumonia, and it was concluded that high-dose VIC could reduce the duration of hospitalization of patients with severe pneumonia by 36%. There was also found to be a lower incidence of pneumonia in people who received vitamin C supplementation [4,126,130,133]

COVID-19 is a new form of pneumonia caused by infection with the SARS-CoV-2 virus, provoking an excessive inflammatory reaction leading to ARDS and multiple organ failure. Some patients may experience mild symptoms, while others may be asymptomatic, but about 5% of cases go on to develop ARDS. In this context, VIC may protect the endothelium against oxidative damage and contribute to the faster healing of damaged tissues. SARS-CoV-2 can suppress the activity of the host immune system, but VIC contributes to enhancing the activity of the immune system by amplifying the antiviral immune response. In addition, VIC has been shown to reduce neutrophil accumulation in the lungs, aiding the clearance of alveolar fluid and strengthening the barrier function of the lung epithelium. The fact that VIC helps decrease the duration and symptoms of respiratory infections may be a key factor in treating the symptoms and progression of COVID-19 [4,126,130,135,136].

### 5.3. Sepsis and Critical Care

Sepsis is a severe medical condition with a high mortality rate (20–35%) among patients, which is characterized by an uncontrolled response of the body to infection, with a systemic inflammatory response, ultimately leading to severe organ dysfunction. The characteristic symptoms of sepsis are the development of systemic inflammation and multiple organ dysfunction up to the development of septic shock. Sepsis can affect any organ system, and one of the most serious complications is ARDS. Sepsis particularly affects patients susceptible to serious infections (e.g., elderly patients with multiple comorbidities), and the most common sources of infection are pneumonia, abdominal infections, and endovascular infections [137,138].

At the cellular level, sepsis produces ROS and RNS that further activate inflammatory transcription factors, affecting the endothelium and microvascularization. Although inflammatory or coagulative mediators have been included in conventional treatment, they have not significantly improved sepsis symptoms and, thus, mortality rates. It has been observed that in critically ill patients, plasma VIC concentration levels are low, which may be associated with a higher incidence of organ failure. This is due to the massive release of cytokines, which affect VIC uptake. At the same time, the low concentration of VIC may also be attributable to the high production of ROS, which contributes to the high VIC uptake [137,138].

Now, there is no optimal treatment that can guarantee the cure of sepsis, and VIC has been proposed as an adjuvant in therapy, in addition to antibiotics, fluids, and vasopressors, due to its ability to reduce inflammation and oxidative stress. The use of VIC could reduce mortality in patients with sepsis. Several placebo-controlled clinical trials focused on VIC administered intravenously in adults with sepsis who were also receiving vasopressor therapy. It was reported that, compared with the group receiving a placebo, the group receiving VIC had a higher mortality rate. A meta-analysis demonstrated the positive effects of VIC administration in patients with sepsis. Two smaller studies also showed decreased mortality in patients treated with VIC. However, the researchers could conclude that adults treated with vasopressors and intravenous VIC had an increased risk of death or organ dysfunction compared to the placebo group [137,138].

### 5.4. Cancer Therapy

The first approach to VIC as a cancer treatment was in 1970 when chemist Linus Pauling and physician Ewan Cameron suggested that a high concentration of VIC administered to patients with terminal cancer could increase survival rates [139,140]. Their pioneering study demonstrated that the daily administration of 10 g of VIC to 100 terminally ill cancer patients significantly improved outcomes, with 10% surviving, compared to no survivors among 1000 patients receiving conventional treatment alone. In more detail, the patients receiving VIC supplementation had a mean survival time of more than 210 days, while the mean survival time of controls was 50 days [141].

VIC has started to be studied to obtain new and efficient treatment in the fight against cancer due to its antioxidant, pro-oxidant, and gene expression regulator properties. These properties help to keep tissues and their functions in better condition by protecting macromolecules (e.g., proteins, fats, DNA) from oxidation [139,140]. VIC is involved in various molecular mechanisms that underlie its anticarcinogenic effect. VIC leads to the inhibition of cell proliferation and growth by generating ROS and altering the expression of genes involved in angiogenesis, glycolysis, and metastasis by regulating the transcriptional activity of hypoxia. VIC also allowed for T lymphocyte infiltration and induced cytokine production, resulting in an anti-tumor immune response [142].

**Figure 4:**
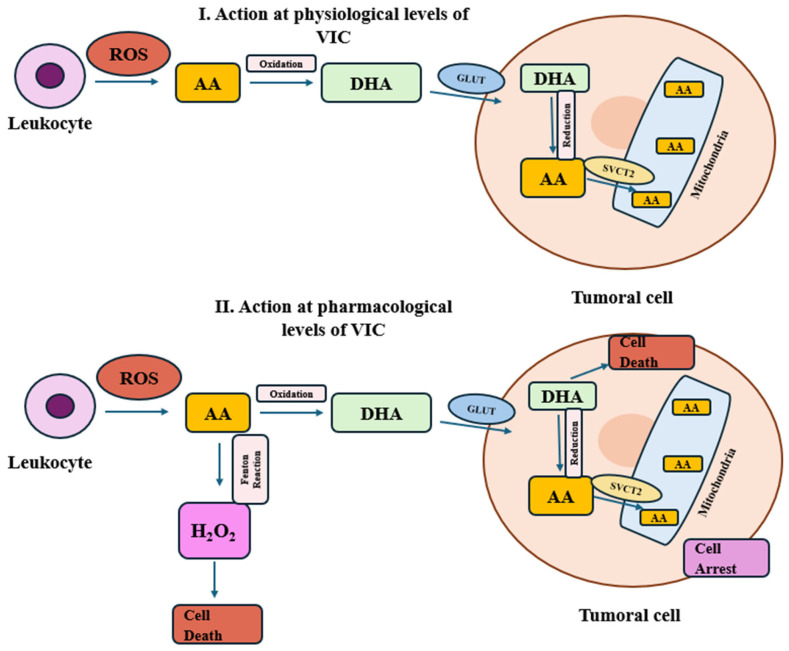
Effects of VIC doses on tumoral cells: I. physiological dose; II. pharmacological dose. Created based on information from [143,148].

As a potential cancer treatment, it has also been observed that the effects of VIC on cancer are dependent on the route of administration, whether oral or intravenous, as well as on the mode of transport of VIC into tumoral cells (Figure 4). For example, it has been observed that some tumor cells absorb more VIC due to increased expression of SVCT2 and/or GLUT1 genes. To be transported, dehydroascorbic acid has to be formed by oxidation of extracellular ascorbic acid to be taken up by the transporter cell. The dehydroascorbic acid is again reduced inside the cell, which consumes other antioxidants and leads to intracellular oxidative stress. Also, in tumors with high SVCT2 gene expression (e.g., breast cancer, liver cancer), VIC killed cancer cells by mega-dose (0.5 mM) administration of VIC [143]. It has also been observed that AA and/or DHA can act as an adjuvant in cancer drug therapies such as paclitaxel [144], cisplatin [145], and methotrexate [146], highlighting the role of GLUT as a carrier for VIC in cancer cells and showing promise for future combined treatment approaches.

Nonetheless, there is still limited evidence and controversy around DHA role in VIC cancer treatment. While some studies suggest a potential role of DHA in cancer therapy, others, such as Piotrowski et al. [147], report no significant cytotoxic effects under clinically relevant conditions. The authors concluded that it was rather the effect of high-dose ascorbate than of DHA that induced severe cytotoxicity in human glioblastoma cell lines. Therefore, further research is required to confirm DHA efficacy and establish its mechanisms of action.

For effective administration of VIC in the body, the route of administration must be carefully considered. Taking into account the barriers related to oral administration, the plasma concentration of VIC is achieved only at μM levels, so intravenous administration of VIC is preferable to achieve a higher concentration. On the other hand, each cancer cell type has its specific ability to absorb and metabolize VIC. For example, breast cancer cell lines had a high capacity to reach the oxidized form of VIC due to overexpression of SVCT2 genes, associated also with a higher resistance to oxidative stress. The use of pharmacologic doses of VIC led to increased sensitivity to treatment. Figure 4 proposes a model of VIC transporters in breast cancer cells. Thus, at physiological doses of ascorbic acid, it is oxidized to DHA by free radicals generated by leukocytes. DHA is then transported into cancer cells by glucose transporters (GLUTs), where it is reduced and returned to the ascorbic acid form, which reduces oxidative stress in the cells. This can give cancer cells a protective effect that supports their survival rate. Instead, high doses of ascorbic acid may generate a higher concentration of free radicals by oxidative reactions. The free radicals enter cancerous cells and produce oxidative stress. In this case, ascorbic acid can induce cancerous cell death and cellular arrest by an intense oxidative process [148].

A study on mice [149] showed that in vivo peritoneal injection of 4 g of VIC per kilogram of body weight conduced to a decreased rate of ovarian, pancreatic, and glioblastoma tumor growth by pro-oxidation effect. The pro-oxidative effect of VIC is associated with the reduction of transitional metals (e.g., Fe^3+^ and Cu^2+^), and hydrogen peroxide’s appearance leads to free radicals formed by Fenton and Haber–Weiss reactions.

Thus, VIC injection leads to extracellular hydrogen peroxide production, which is further accumulated in the mitochondria of cancer cells, therefore inducing caspase-independent autophagy. This pro-oxidant effect has been observed in K-ras and BRAF cancer cells. It was also observed that a 20 mM VIC concentration could selectively kill cancer cells without affecting healthy cells [143,148].

In a study led by Gillberg et al. [142], oral administration of VIC in patients with myeloid cancer was investigated to determine whether supplementation can lead to improvement of VIC deficiency and its effects on treatment. Patients with hematologic malignancies are generally deficient in VIC. In their case, VIC is essential for the TET-induced conversion of 5-methylcytosine (5 mC) to 5-hydroxymethylcytosine (5 hmC), the first step in active DNA demethylation. Thus, the administration of 500 mg VIC led to the restoration of plasma VIC levels in all the patients to which it was administered. The researchers observed an increase in the 5 hmC/5 mC ratio compared to placebo-treated patients, which may enhance the biological effects of DNMTis. It also suggests that administering VIC to DNMTis should be studied further in larger clinical trials.

Some recent statements showed that VIC has a synergic effect combined with some anti-tumoral drugs. For example, gemcitabine combined with VIC has a cytotoxic effect against eight pancreatic cancer cell lines by producing hydrogen peroxide. Also, in a mouse model, it was observed that this combination inhibited tumor growth. Also, the same effect was observed in the administration of carboplatin and paclitaxel in combination with VIC on ovarian cancer cell lines. VIC also reduced the adverse effects of chemotherapy treatment in mouse models [148,150,151]. A 2018 study [151] showed the effect of auranofin with VIC and a strong anticarcinogenic effect of these two combined against MDA-MB-231 and other breast cancer cell lines. Researchers concluded that this combination has the potential to be an efficient treatment for triple-negative breast cancer and other cancer types with the same redox potential. Another in vitro study [152] has shown that high-dose vitamin C (1.25 to 20 mM) combined with conventional cancer treatments could boost the effects against breast cancer. On the other hand, a study conducted by Wang et al. [153] investigates the efficacy of adding VIC to the treatment regimen of patients with unresectable metastatic colorectal cancer (mCRC). The 442 patients were divided into two groups: an experimental group receiving high-dose VIC + FOLFOX + bevacizumab, and a control group receiving FOLFOX + bevacizumab alone. Treatment was given in two two-week cycles for 12 weeks, followed in some cases by maintenance treatment. The study showed that VIC added at a high dose (1.5 g/kg/day, intravenously for 3 h from day 1 to day 3) did not significantly contribute to overall benefit. However, the treatment was well tolerated by patients and may offer benefits for patient groups with RAS mutations. A phase II trial, realized by Paller et al. [154], investigated the effect of high-dose intravenous VIC combined with docetaxel in men with progressive metastatic castration-resistant prostate cancer. In this respect, 47 patients were randomized 2:1 to receive docetaxel (75 mg/m^2^) intravenous and high-dose VIC (1 g/kg) or placebo. The study aimed to observe the PSA50 response and the side effects. The researchers concluded that high-dose VIC and docetaxel did not improve the PSA50 response, toxicity, or other clinical outcomes.

Yet, VIC showed that it can reduce the adverse effects of radiotherapy treatment. VIC can enhance the cytotoxicity effect of the radiation in different tumoral pancreatic cell lines without affecting healthy cells. This effect is produced because of increased oxidative stress in tumoral cells. In this context, VIC led to a decreased tumor growth rate and a higher survival rate. It can be concluded that VIC can be used as a complementary treatment for radiotherapy for pancreatic cancer patients [148,155].

Thus, it has been shown that using VIC in high doses can exert anti-tumor activity by generating ROS in malignant cells without affecting healthy cells. It has also been suggested that VIC can enhance the effects of both chemotherapy and radiotherapy [147,156]. However, studies are still needed to demonstrate the efficacy and safety of using high doses of VIC for such a therapeutic application.

### 5.5. Wound Healing

The skin is the largest organ of the human body, composed of the epidermis (epithelial tissue), dermis (connective tissue), and the subcutaneous layer called hypodermis. The epidermis comprises multiple layers, such as the stratum corneum, the stratum granulosum, the stratum spinosum, and the basal stratum, each of which is composed of cells such as keratinocytes, pigment cells and melanocytes, Langerhans cells, mast cells, and Merkel cells. The dermis has papillary and reticular layers that contain fibroblasts, which are important in the synthesis of collagen, elastin glycosaminoglycans, blood vessels, neovessel endings, sweat, and sebaceous glands, including hair bulbs. The hypodermis consists of adipose cells that form fat lobules. The skin is involved in protecting the body from external factors (e.g., chemicals, pathogens), moisturizing, temperature regulation, and vitamin D synthesis [157,158].

Wound healing assumes numerous stages, such as hemostasis, inflammation, proliferation, and remodeling. Hemostasis is the first stage in wound healing and is accomplished by constricting blood vessels and forming fibrin clots to stop bleeding, followed by inflammation. In inflammation, immune cells (e.g., neutrophils and macrophages) arrive at the wound’s site and help eliminate pathogens and cell debris. In the proliferation stage, fibroblasts and keratinocytes migrate and proliferate at the wound site, forming collagen fibers and regenerating the injured tissue; then, in the remodeling stage, collagen is reorganized, and thus the structure and functions of the skin are restored [159]. In this regard, VIC plays an essential role in synthesizing connective tissue, especially in synthesizing collagen, for which it plays a cofactor role. VIC may also confer strength to collagen fibers, especially tensile strength. It has also been observed that VIC may contribute to increased activity of dermal fibroblasts, potentiating the wound healing effect. In addition to its role in collagen synthesis, VIC may reduce the occurrence of pro-inflammatory factors during wound healing, accelerate the healing process, and contribute to the faster removal of neutrophils from the inflamed area. Hypoxia-induced inhibitory factor-1 (HIF-1), essential for cellular metabolism and angiogenesis contributor, may also be activated in wounds. Under hypoxic conditions, this factor is no longer adequately degraded and can form protein complexes that lead to the transcription of genes responsible for cell proliferation, angiogenesis, and glucose metabolism. In the presence of VIC, this factor reduces its activity, helps combat the negative effects of hypoxia, and considerably reduces scarring [158,160,161,162].

For a better effect on wound healing, it has been observed that topically applied VIC may be more effective for delivery to the skin than orally administered, and there is an increased improvement in tissue regeneration and healing of surgical wounds. Other studies have also observed that administering VIC to diabetic mouse models contributed to increased collagen production, improved angiogenesis, and reduced inflammation. In vitro on human fibroblast cells, VIC was found to increase the expression of type I and III collagen [158,160,161].

Other studies were performed by Bikker et al. [162] on hospitalized patients. The first study was performed on a case represented by a 59-year-old man who underwent a laparotomy for diverticulitis, followed by appendectomy and sigmoid colectomy. It was noted that after surgery, she had had two abdominal dehiscences, which were surgically corrected. Also, the healing process was slow, and the size of the surgical wound was not reduced after alginate dressings were applied. Thus, oral supplementation with VIC (1000 mg/day) was started, and after about 3 weeks, healthy granulation was observed, with a considerable reduction in the wound, which closed completely after almost 3 months.

Another case [162] was a 79-year-old woman with severe ulcers on the lower limbs, followed by surgical resection of the necrosis, after which slow healing was observed. After supplementation with 1000 mg/day of VIC, the wounds started to heal, and after 8 weeks, healing was complete. Another 68-year-old woman with Crohn’s disease and diabetes underwent ileocecal resection and developed severe peritonitis. After administration of 1000 mg/day of VIC, both intravenous and orally, healing was accelerated without the need for surgery.

Another study [163] aimed to evaluate the effects of VIC supplementation on the healing process of wounds caused by dental extractions. Thus, patients were given oral doses of 600 mg/day of VIC and 1500 mg/day divided over three doses for 10 days. A dose of 600 mg/day led to a significant reduction in the wound size 7 days after extraction, compared with a dose of 1500 mg/day, which did not provide any additional benefit. VIC was found to lead to rapid fibroblast differentiation and granular tissue formation. It was also observed that postoperative pain was reduced due to the reduced inflammatory process due to VIC.

An analysis of several studies [164] on using VIC in treating foot ulcers in patients with diabetes suggested that administering VIC accelerated healing. Patients who received VIC had a significant reduction in healing time, while it also led to reduced postoperative pain. Likewise, another analysis [165] evaluated the potential of VIC against leg ulcers, noting that VIC supplementation led to good healing outcomes. The same review evaluated the possibility of using VIC in oral care, recommending its supplementation in patients’ diets [165].

## 6. Ongoing Research

According to the ClinicalTrials.gov platform, more studies could bring promising new results regarding the use of VIC as a potential therapeutic agent. Thus, selected interventional trials with results in phases 3 and 4 are presented in Table 3.

## 7. Conclusions

Vitamin C is essential for maintaining biological functions through its antioxidant effect and mode of action as a cofactor for enzymes involved in synthesizing collagen or catecholamines. It is vital to prevent scurvy, which is associated with a major deficiency of VIC in the body. At the same time, VIC is important in maintaining long-term health, with a multitude of benefits that may contribute to the prevention of chronic disease, cardiovascular disease, and even cancer prevention or treatment.

Thus, this review highlighted aspects related to the therapeutic properties of VIC. Some of the studies presented in this review demonstrate the beneficial effects of administering VIC for various conditions (e.g., scurvy, cardiovascular disease, hypertension, infections, pneumonia, COVID-19, sepsis, neurodegenerative diseases, wound healing), as well as data that contradict these aspects or that do not find an association between VIC and the presenting conditions. In some cases, researchers have seen the benefits of using VIC in therapy, while other results have indicated that VIC may increase the risk of certain diseases. Discrepancies in study results may arise from differences in monitoring plasma Vitamin C levels, varying delivery methods (oral vs. intravenous), and challenges in controlling external variables such as diet. An important factor in this is the lack of long-term studies that focus on the concentration of VIC in the blood, not just on a dietary questionnaire. It has also been observed that VIC is not efficiently absorbed in high concentrations but may produce undesirable adverse effects, such as kidney stones or nephropathy oxalate. Targeted delivery systems, such as encapsulated vitamin C or controlled-release formulations, could minimize gastrointestinal side effects associated with high-dose supplementation. Hence, future studies should focus on standardizing vitamin C plasma monitoring protocols, optimizing delivery systems to maintain therapeutic levels, and exploring combined antioxidant therapies to enhance efficacy.

In conclusion, with continuous studies that deepen the understanding of its effects and optimum dosages, VIC has the potential to become an alternative therapeutic strategy, remaining an important nutrient for both health maintenance and biological function in the human body.

## Figures and Tables

**Table 1:** Recommended Dietary Allowances (RDAs) for VIC (mg). Adapted from [66].

Age	0–6 Months	7–12 Months	1–3 Years	4–8 Years	9–13 Years	14–18 Years	19+ Years
**Male**	40	50	15	25	45	75	90
**Female**	40	50	15	25	45	65	75
**Pregnancy**	-	-	-	-	-	80	85
**Lactation**	-	-	-	-	-	115	120
**Smokers**	35 mg more than non-smokers						

**Table 2:** Tolerable Upper Intake Levels (ULs) for VIC (mg). Adapted from [66].

Age (Years)	1–3	4–8	9–13	14–18	19+
**Male**	400	650	1200	1800	2000
**Female**	400	650	1200	1800	2000
**Pregnancy**	-	-	-	1800	2000
**Lactation**	-	-	-	1800	2000

**Table 3:** Summary of interventional clinical studies about VIC as a therapeutic agent.

ClinicalTrials. gov ID	Official Title	Conditions	Intervention/Treatment	Phase	Ref.
NCT04328961	Efficacy of Hydroxychloroquine for Post-exposure Prophylaxis (PEP) to Prevent Severe Acute Respiratory Syndrome Coronavirus 2 (SARS-CoV-2) Infection Among Adults Exposed to Coronavirus Disease (COVID-19): a Blinded, Randomized Study	COVID-19 Corona Virus Infection SARS (Severe Acute Respiratory Syndrome) SARS-CoV-2	Drug: Hydroxychloroquine Sulfate Drug: Ascorbic Acid	Phase 2 Phase 3	[166]
NCT03509350	A Multi-center, Randomized, Placebo-controlled, Double-blind, Adaptive Clinical Trial of Vitamin C, Thiamine and Steroids as Combination Therapy in Patients With Sepsis.	Sepsis	Drug: Vitamin C Drug: Thiamine Drug: Hydrocortisone Drug: Vitamin C Placebo Drug: Thiamine Placebo Drug: Hydrocortisone Placebo	Phase 3	[167]
NCT03389555	Ascorbic Acid, Hydrocortisone, and Thiamine in Sepsis and Septic Shock-A Randomized, Double-Blind, Placebo-Controlled Trial	Sepsis Septic Shock Metabolic Disturbance	Drug: vitamin C, vitamin B1, hydrocortisone Drug: Normal saline	Phase 2 Phase 3	[168]
NCT03338569	Evaluating Vitamin C in Septic Shock: A Randomized Double Blind Placebo Controlled Trial	Septic Shock Sepsis	Drug: Vitamin C Drug: Placebo	Phase 2 Phase 3	[169]
NCT02956057	A Head-to-head Comparison of Efficiency and Tolerance of 4-L Polyethylene Glycol and Sodium Picosulphate/Magnesium Citrate, Polyethylene Glycol/Ascorbate Before Colonoscopy	Colonoscopy	Drug: Polyethylene Glycols Drug: Natrium picosulfate/Magnesium citrate Drug: Polyethylene glycol /Ascorbic acid	Phase 4	[170]
NCT02209636	Phosphate Lowering to Treat Vascular Dysfunction in Chronic Kidney Disease	Chronic Kidney Disease Cardiovascular Disease	Drug: Lanthanum carbonate Drug: placebo Drug: Ascorbic Acid Drug: Nitroglycerin Procedure: Flow-mediated dilation measurement Procedure: Aortic pulse-wave velocity Procedure: Endothelial cell collection	Phase 4	[171]
NCT02175212	Phase III Multicenter Randomized Trial of Adjuvant Androgen Deprivation in Combination With Three-dimensional Conformal Radiotherapy Doses in High and Intermediate Risk Localized Prostate Cancer.	Prostate Adenocarcinoma	Drug: Short-term androgen deprivation Drug: Long-term androgen deprivation Radiation: Short-term androgen deprivation Radiation: Long-term androgen deprivation	Phase 3	[172]
NCT01723696	Vitamin C to Decrease Effects of Smoking in Pregnancy on Infant Lung Function	Pulmonary Function; Newborn, Abnormal Infant Wheeze In utero Nicotine Secondhand Smoke	Dietary Supplement: Vitamin C + prenatal vitamin Dietary Supplement: Placebo tablet + prenatal vitamin	Phase 2 Phase 3	[173]
NCT01595516	Nebivolol and Endothelial Regulation of Fibrinolysis	Prehypertension Hypertension	Drug: Nebivolol Drug: Metoprolol Drug: Placebo Other: Bradykinin Other: Saline Other: Vitamin C	Phase 4	[174]
NCT01423162	Iron Bioavailability Study of Fortified Oat Drink	Iron Absorption	Other: Dietary Intervention (with Vit C then without Vit C) Other: Dietary Intervention (without Vit C followed by with Vit C)	Phase 4	[175]
NCT01413360	Phase 4 Study of High Dose Vitamin C in Chronic Hepatitis Patients	Chronic Hepatitis Chronic Hepatitis C Chronic Alcoholic Hepatitis	Drug: High dose vitamin C	Phase 4	[176]
NCT01332578	A Comparison of Solid and Soluble Forms of Cold and Influenza Remedies	Influenza Common Cold	Drug: Paracetamol Drug: Phenylephrine Drug: Ascorbic Acid	Phase 4	[177]
NCT01167569	Double-Blind, Randomized Study of High Dose Vitamin C On Outcome in Cardiac Surgery Patients	Reperfusion Injury	Drug: Ascorbic Acid Other: 5% Dextrose Water or Normal Saline	Phase 4	[178]
NCT01160198	A Multicentre, Randomized, Laboratory-blinded, Parallel-group Study to Demonstrate the Efficacy and Tolerability of Ferrous Bisglycinate Chelate in Iron Deficiency Anaemia and to Compare These with Those of Ferrous Ascorbate.	Hematopoiesis	Drug: ferrous ascorbate Dietary Supplement: ferrous bisglycinate chelate 1 OD Dietary Supplement: ferrous bisglycinate chelate 2 OD	Phase 3	[179]
NCT01107730	Randomized Double Blind Study of Administration of Vitamin C for Prophylaxis of Post-operative Atrial Fibrillation in On-pump Cardiac Surgery Procedures	Atrial Fibrillation	Drug: Vitamin C Drug: L-Carnitine Drug: Placebo	Phase 3	[180]
NCT00953212	A Randomized Controlled Trial to Compare Prophylaxis With Oral Ascorbic Acid, Oral Amiodarone or Both in Combination With Beta Blockers to Reduce Postoperative Atrial Fibrillation After Cardiac Surgery	Atrial Fibrillation Atrial Flutter	Drug: beta blockers Drug: amiodarone Drug: ascorbic acid	Phase 3	[181]
NCT00860470	Antenatal Multiple Micronutrient Supplementation to Improve Infant Survival and Health in Bangladesh	Infant Mortality Preterm Birth Low Birth Weight Neonatal Mortality Perinatal Mortality	Dietary Supplement: Iron (27 mg)–folic acid (600 ug) Dietary Supplement: Multiple micronutrient	Phase 3	[182]
NCT00135707	A Randomized Clinical Trial of Antioxidants to Prevent Preeclampsia and An Observational Cohort Study to Predict Preeclampsia	Preeclampsia	Drug: Dietary Supplement/Vitamins Drug: Placebo for Vitamin C and Vitamin E	Phase 3	[183]
NCT00064753	Folic Acid for Vascular Outcome Reduction in Transplantation (FAVORIT)	Chronic Kidney Disease Cardiovascular Disease Death	Drug: High-Dose Multivitamin Device: Low-Dose Multivitamin	Phase 2 Phase 3	[184]
